# Clinical application of the experimental ADL test for patients with cognitive impairment: pilot study

**DOI:** 10.1038/s41598-020-78289-z

**Published:** 2021-01-11

**Authors:** Yong-Hyun Lim, Yookyeong Baek, Soon Ju Kang, Kyunghun Kang, Ho-Won Lee

**Affiliations:** 1 Center of Self-Organizing Software-Platform, Kyungpook National University, Daegu, South Korea; 2 Department of Neurology, School of Medicine, Kyungpook National University, 80 Daehakro, Bukgu, Daegu, 41566 Korea; 3 School of Electronics Engineering, College of IT Engineering, Kyungpook National University, Daegu, South Korea; 4 Brain Science and Engineering Institute, Kyungpook National University, Daegu, South Korea

**Keywords:** Diseases, Neurological disorders, Diagnostic markers

## Abstract

We employed a hospital-based Internet of Things (IoT) platform to validate the role of real-time activities of daily living (ADL) measurement as a digital biomarker for cognitive impairment in a hospital setting. Observational study. 12 patients with dementia, 11 patients with mild cognitive impairment (MCI), and 15 cognitively normal older adults. The results of 13 experimental ADL tasks were categorized into success or fail. The total number of successful task and the average success proportion of each group was calculated. Time to complete the total tasks was also measured. Patients with dementia, patients with MCI, and cognitively normal older adults performed 13 experimental ADL tasks in a hospital setting. Significant differences in the average success rate of 13 tasks were found among groups. Dementia group showed the lowest success proportion (49.3%) compared with MCI group (78.3%) and normal group (97.4%). Correlation between classical ADL scales and the number of completed ADL tasks was statistically significant. In particular, instrumental ADL (I-ADL) had stronger relationship with the number of completed ADL tasks than Barthel’s ADL (B-ADL). Dementia group required more time to accomplish the tasks when compared to MCI and normal groups. This study demonstrated that there is a clear relationship between the performance of experimental ADL tasks and the severity of cognitive impairment. The evaluation of ADLs involving the IoTs platform in an ecological setting allows accurate assessment and quantification of the patient’s functional level.

## Introduction

Activities of daily living (ADL) are utilized routinely as predictors of health and function among community-dwelling older adults to detect the early onset of disability to enable appropriate care management. Currently, the approaches for assessment are inadequate, lacking reliability, sensitivity, and validity. It is, therefore, critical to have instruments that would be more sensitive in detecting preclinical stages of functional decline.

Accomplishing routine ADLs requires several fundamental abilities: cognitive, motor, and perceptual abilities^1^. Among these abilities, impairment in cognitive function hinders overall performance of ADLs of older adults, which could interrupt with their independent living^2^. Therefore, evaluating the decline in ADLs is crucial for cognitively impaired patients to enable assessment of their actual functional status and decide whether a patient would need assistance with daily tasks. Besides, the accurate assessment of ADLs contributes to the diagnosis of cognitive disorders, including dementia and mild cognitive impairment (MCI). Longitudinally, changes in ADL performance could be monitored through the course of the cognitive disorders to investigate the progression or improvement with medications.

Classical ADL assessment relies on patient-reported outcomes in which the patients provide responses regarding their statuses themselves or indirect ratings from informants such as caregivers. However, considering patients’ old age and cognitive dysfunction, the accuracy and reliability of self-rated questionnaires are doubtable^3^. Furthermore, previous research has proven a disagreement between indirect ADL ratings from caregivers and the actual functional statuses of patients^4,5^. Caregivers tend to overestimate their ADL assistance and report lower levels of the functional status of patients, compared with patients’ direct assessment; thus, indicating that the indirect measurement using questionnaires has unequivocal limitations in that the subjective evaluation of ADLs is susceptible to bias and errors.

Several studies have attempted direct measurement of ADLs in a home or experimental settings^6–9^. The use of sensor-based technology for the recognition and classification of ADLs at home environment has been proposed in many studies^9–11^. Some of them used body-mounted systems that allow data quantitation, along with disease risk and early detection, while others utilized non-intrusive ambient sensor systems that enable capturing of environmental data for cumulative measurements. These evaluations have proven that those with body-mounted sensors, as well as non-intrusive ambient sensors could distinguish and classify different ADLs successfully by detecting and quantifying patients’ behaviors. Furthermore, it has been shown that differences in ADL pattern-performance between dementia patients and healthy controls are detectible with the use of unobtrusive in-home sensors^12^. On the other hand, there have been attempts to measure ADLs by direct observation and performance quantification of ADL tasks in a laboratory environment^6^. Assigning identical tasks to every participant in a controlled setting provides more objective and reliable data regarding the performance of ADLs.

In light of a need for direct measurement of ADLs, it is highly desirable to assess ADLs with real-time observations. On top of that, it is essential to control different variables which can exist in the living space by implementing an experimental space where ADLs can be evaluated. And unlike previous studies that focused on observing and categorizing various ADL performances, we made it possible to compare cognitive functions in a more objective way by having all participants perform the same type of ADLs.

We employed a hospital-based Internet of Things (IoT) platform to validate the role of digital real-time ADL measurement for cognitive impairment, as the next steps in the progress of the development of the IoT devices. In the course of the advancement of this IoT instrument, there have been many studies on various platforms for the recognition of daily activities in the living environment and/or monitoring the deterioration of cognitive abilities^13–17^. While video- or microphone-based platforms are remarkable in detecting human activities technically, concerns over maintaining privacy have been raised. Installing videos and microphones in the most private space and transmitting the information on daily lives to an external server are likely to fail approval by the general population, despite their technological advantages.

Our IoT instruments platform was constructed with deliberation on the optimal balance between privacy and technical feasibility. A series of processes, including distinguishing a person’s identity, deciding whether a person’s behavior is in the normal range or not without videos or microphones, and transmitting the information to caregivers or a designated community care center requires highly sophisticated technologies.

We hypothesize that there would be considerable differences in the performance of the experimental tasks depending on the degree of cognitive impairment of the study participants. At the same time, we explored the potential of the hospital-based IoT platform in assessing ADLs for clinical decision-making. With the results from this platform, we analyze the discriminant power of the experimental tasks to determine if the real-time measurement of ADLs would have clinical significance in distinguishing the cognitively impaired patients.

## Results

### Analysis of the task success proportion and task completion time

**Figure 1:**
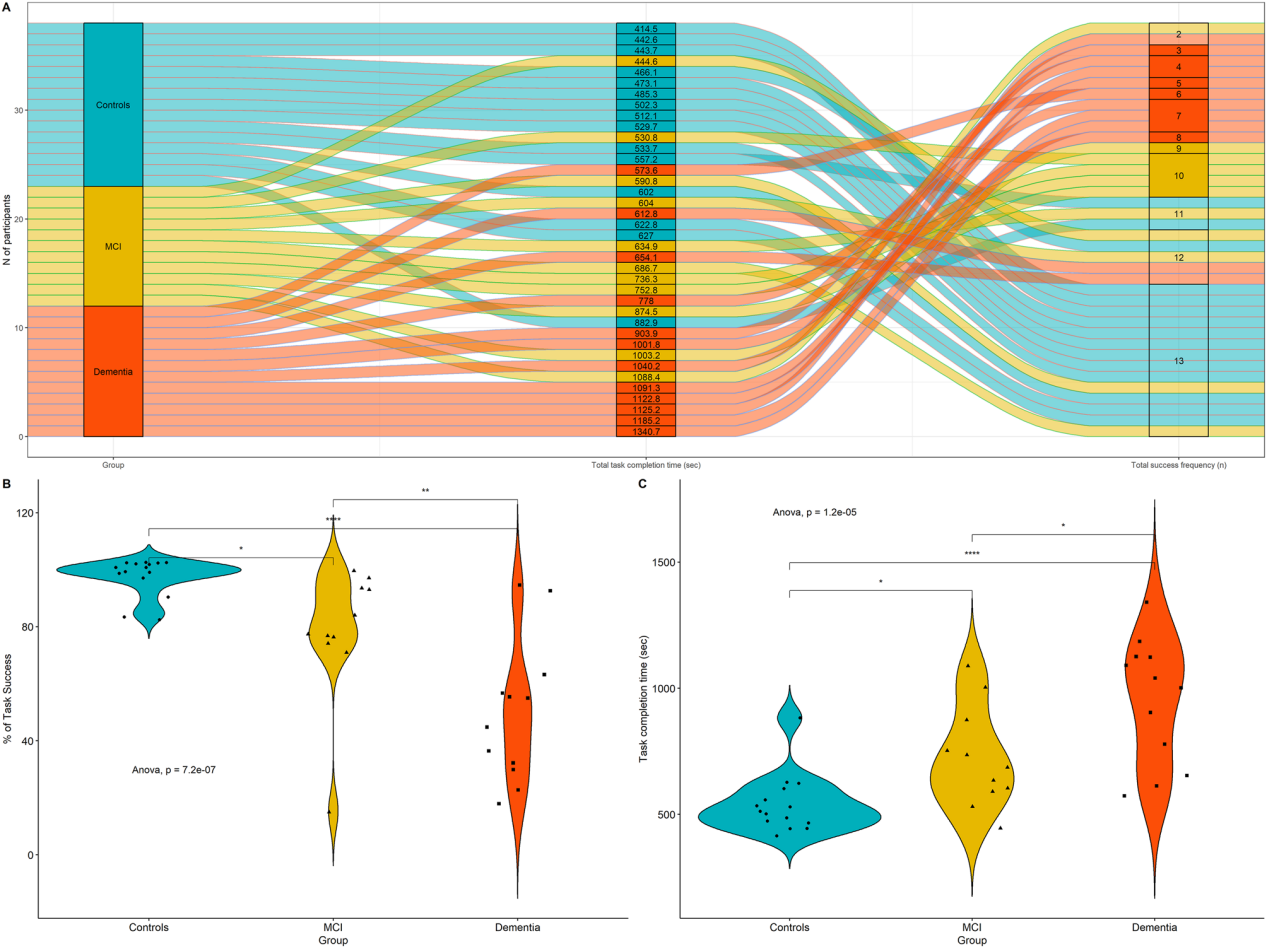
Alluvial diagram and group difference for the task activities. **(A)** Alluvial diagram showing the total completion time and total success frequency of each group and all participants as a flow of data. **(B)** Group difference in task success rate. **(C)** Group difference in task completion time.

Figure 1 shows the success proportion of the thirteen tasks. The average success proportions of the normal control group, MCI group, and the dementia group were 97.4%, 78.3%, and 49.4%, respectively (95% confidence interval [CI_*controls*_] = 87.56%–107.31, CI_*MCI*_ = 66.79%–89.85%, CI_*dementia*_ = 38.32%–60.40%; *P* < 0.001). Among the three groups, the dementia patients showed the lowest average success proportion. Overall, among the 13 different tasks that were subjected to completion, the type of task that involved using a microwave oven (task 12) demonstrated the lowest success proportion while entering the test room (task 1) demonstrated the highest success. In the analysis of task completion time, there were significant differences among the three groups regarding the time it took to complete the tasks (95% CI_*controls*_ = 34.89–50.47, CI_*MCI*_ = 47.02–64.71, CI_*dementia*_ = 62.65–80.43; *P* < 0.001). On average, the dementia group required more time to complete the tasks, compared with the MCI group (95% CI = 3.03–28.32; *P* < 0.05) and normal control group (95% CI = 16.61–41.10; *P* < 0.001). Among the tasks, moving to the kitchen (task 9) required the longest amount of time, while entering the test room took the least amount of time (task 1). In summary, dementia patients needed more time to finish the tasks and yet showed the least successful performance, whereas the normal group performed most of the tasks successfully with the least time.

Figure 1A shows both the total task completion time and the total number of successful tasks for all participants. As shown in Fig. 1 B-C, the task success proportions and task completion time of the dementia group turned out to vary in distribution among the patients. On the other hand, the normal group demonstrated a more homogeneous distribution pattern with the success proportions and the time it took to complete the tasks. The MCI group exhibited an in-between distribution pattern, being more variable than that of the normal group but more homogeneous than that of the dementia group. These distribution patterns of the three assessment groups indicate that there were systematic differences in task performances among them, which correlated with each group’s cognitive function.

### Linear discriminant analysis (LDA) for the digitalized ADL task

Table 1 shows LDA conducted based on the performance of each participant group. Three models were constructed with the independent variables, which consisted of criteria for distinguishing each cognitive group, and the group variables, which were three cognitively different groups. Model 1 consisted of the scores from clinical scales, including Korean version of Mini-Mental State Examination (K-MMSE), Korean version of Expanded Clinical Dementia Rating scale (CDR), Global Deterioration Scale (GDS), Barthel Index Activity of Daily Living (B-ADL), and the Korean version of Instrumental Activity of Daily Living (I-ADL), and Model 2 only included scores from B-ADL and I-ADL. Lastly, Model 3 employed the results of 13 experimental tasks. The classifiers were compared with each other with regards to how effectively a model could classify the participants according to their cognitive level. Linear discriminant function 1 (LD 1) and 2 (LD 2) were extracted from each model. The values from LD 1 of Wilks’ lambda were lower than those from LD 2 of Wilks’ lambda. In contrast, the values from LD 1 of Eigenvalues were higher than those from LD 2 of Eigenvalues. Moreover, the values of LD 1 in all the three models were statistically significant (Model 1; *χ*^*2*^ = 101.84, *P* < 0.001, Model 2; *χ*^*2*^ = 36.58, *P* < 0.001, Model 3; *χ*^*2*^ = 68.39, *P* < 0.001). Model 1 showed the highest discriminant power (canonical *r* = 0.96, *SVD* = 13.6), as this model comprised the individual variables which had been used to discriminate against the experimental groups in the first place. Model 2, which was constructed with B-ADL and I-ADL, excluded the cognition assessment scales (MMSE, CDR, GDS) in order to compare with Model 3, which showed the lowest discriminant power among the classifying groups (canonical *r* = 0.81, *SVD* = 5.8). The misclassification rate of Model 2 was highest among the three models (31.5%). Although Model 3 revealed lower discrimination hit ratio, compared with Model 1, it showed a higher discriminant hit ratio (89.5%) than Model 2 (67.6%).

**Table 1:** Model comparison of linear discriminant analysis.

Model	Wilks’ lambda		Eigenvalues (canonical correlations)		SVD^a^		Discriminant hit ratio (%)
	LD1	LD2	LD1	LD2	LD1	LD2	
Model 1	0.04^***^	0.51^***^	11.24 (0.96)	0.97 (0.70)	13.6	4.2	100%
Model 2	0.34^***^	0.99	1.94 (0.81)	0.01 (0.11)	5.8	0.5	67.6%
Model 3	0.09^***^	0.66	5.96 (0.93)	0.52 (0.58)	10.02	3.1	89.5%

*LD1* linear discriminant function 1, *LD2* Linear discriminant function 2, *Model 1* MMSE + CDR + GDS + B-ADL + I-ADL, *Model 2* B-ADL + I-ADL, *Model 3* task 1 + task 2 + … + task 13. Class variable(or target variable) of all models was group, *SVD* singular value decomposition-compute ‘lda function’ of R MASS package.

^***^p < 0.001.

**Figure 2:**
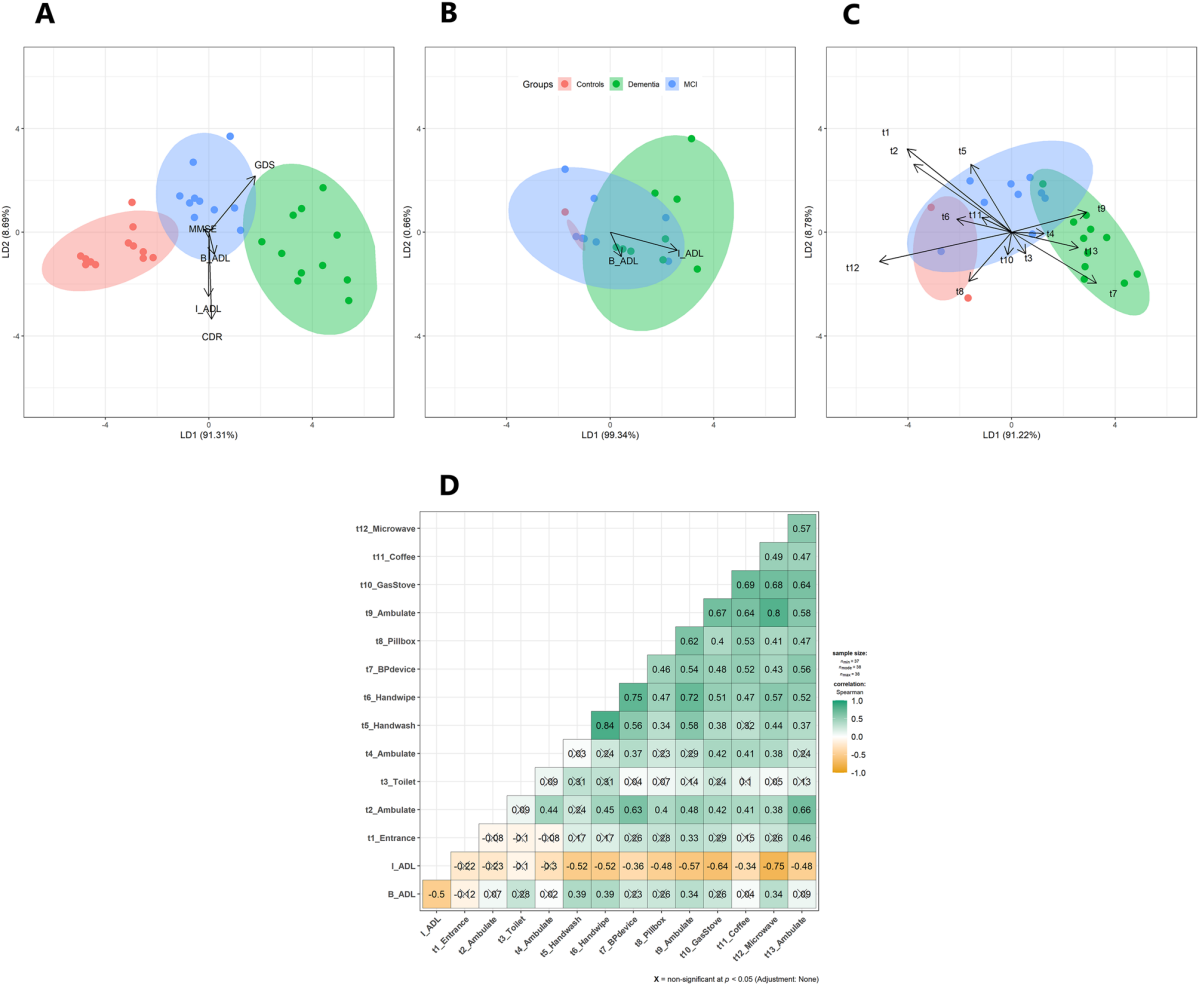
The results of LDA and correlation analysis. **(A)** Biplot of LDA with 5 clinical scales as predictors(MMSE, CDR, GDS, B-ADL, I-ADL). **(B)** Biplot of LDA with 2 ADL clinical scales as predictors(B-ADL, I-ADL). **(C)** Biplot of LDA with 13 experimental ADL tasks used in this study(“t1” = task1. predictor variables; task1 + task2 + …. + task13). **(D)** The Spearman's correlation between the total task success frequency and the clinical ADL scales.

Figure 2A–C represents how the LDA classifier was utilized to classify the cognitively different participants into three groups using standardized coefficients of linear discriminants. The larger the coefficient, the more significant the contribution of each independent variable in the group’s discrimination.

### The relationship between the clinical ADL scores and the number of successful tasks

An association between the scores obtained on the clinical ADL scales (B-ADL and I-ADL) and the results of 13 experimental tasks was identified. Figure 2D reveals that I-ADL scores of participants significantly correlated with the performance of nine tasks (t4; hand washing, t5; hand wiping, t7; measuring blood pressure, t8; using pillbox, t9; ambulating, t10; using a gas stove, t11; using a coffee machine, t12; using a microwave oven, t13; ambulating). In particular, the results of using microwave oven (task 12) and the gas stove (task 10) showed the strongest correlation with I-ADL scores (*rho* =  − 0.75; *rho* =  − 0.64, *P* < 0.05, respectively), followed by the result of ambulating (task 9; *rho* =  − 0.57, *P* < 0.05). On the other hand, B-ADL scores were significantly related to only four of the tasks (t4; hand washing, t5; hand wiping, t9; ambulating, t12; using microwave oven).

## Discussion

The goal of this study was to evaluate the ADL of normal older adults and cognitively impaired patients using IoT devices and network technologies and to verify their effectiveness in the hospital setting. ADL performance of three cognitive groups of dementia patients, MCI patients, and normal participants was evaluated using 13 experimental tasks. We used an IoT-based platform to measure participants’ task success proportion and task completion time and statistically confirmed how effectively these results could classify the groups. Accordingly, the ADL experimental tasks among patients with dementia had the lowest success proportion and the longest completion time of task time. When it came to the distribution pattern of the results, the greater the severity of the group’s cognitive decline, the more heterogeneous the distribution pattern of the individual patient was. Additionally, the classifier composed of the ADL experiment tasks showed greater discriminant power to distinguish three cognitive groups than the classifier composed of B-ADL and I-ADL scores.

Through this experiment, it was evident that there are systemic statistically significant differences in the performance of ADL tasks relative to the different cognitive levels of the three groups. The dementia group showed the worst performance among the three groups, while the MCI patients showed better performance than the dementia patients but not as good as the normal participants. Thus, the results of performing the experimental tasks, which are derived from ADLs in real life, are likely to reflect the deterioration of patients with cognitive impairment in their living environment.

Our analysis(LDA) that looked at how well the experiments in this study could distinguish the groups which had already been separated by clinical scales. It was proved that the classifier created using the results of the experimental task distinguished the clinical groups to a precise degree, although the task of evaluating the cognitive function itself was not included in the experiment. These results are consistent with the purpose of this study, set to quickly and accurately measure the ability to perform ADLs in a clinical scene by recognizing the success or failure of the task using IoT devices. Moreover, the results of this study not only show the technical feasibility and clinical usefulness of the IoT technology but also suggest the potential of the IoT devices to advance in their development into a useful platform that could evaluate ADLs accurately, while satisfying the conditions to protect the privacy of patients. The advantage of such privacy protection can lead to the use of the IoT devices as a method to measure ADLs in the daily life of patient.

Among the different types of ADL, it has been demonstrated that the decline in I-ADL is more sensitive to MCI or early dementia than B-ADL because I-ADL deteriorates when higher cognitive function is impaired^18–21^. The experimental tasks in this study included both types of ADL tasks; those involving ambulating and maintaining hygiene represent B-ADL, and those involving utilization of different instruments (sphygmomanometer, Smart Pill Reminder, coffee machine, gas stove, and microwave) represent I-ADL. The fact that the performance results of the tasks have a stronger correlation with I-ADL than the B-ADL suggests that the tasks in this study could better detect the deterioration of early cognitive function in a like manner as I-ADL.

The major limitation of this study trace to the experimental procedures because participants with hearing impairment or gait disturbance were excluded who might have difficulties in performing the tasks successfully despite normal cognitive function. Meanwhile, since the tasks of evaluating ADLs are limited to a few, it could not fully represent activities in daily life consisting of hundreds of older adults. In the same vein, evaluations of social activities, outings, and financial management have not yet been incorporated into the tasks due to constraints in the laboratory setting. Also, unlike previous studies in which ADL was assessed over a period of time^12,22^, this was a point-in-time assessment and possibly not fully demonstrate the participants’ daily living skills in their homes or a facility.

Regarding the IoT platform, despite its several advantages, which include the short duration of measurement time, high-level of patients’ privacy, and autonomous network system among the devices, the platform exhibited a relatively high frequency of errors during the experiment (12.15%), and thus, needs to be improved in future studies.

In conclusion, the evaluation of ADLs using the IoT platform demonstrated a clear correlation between cognitive function and ADL performance. The results of ADL performance showed a substantial determinant power in regard to classify the participants into three cognitive groups. The significant association between I-ADL score and ADL performance also suggests that the direct ADL measurement can prove its value in detecting early stage of cognitive decline. Furthermore, the measurement of ADLs in a hospital setting showed the potential to provide clinicians with evidence to understand the cognitive level of patients and tools to follow up on changes in cognitive function in the course of the patient’ disease.

## Method

### Participants

We conducted an observational study with an enrollment of a total of 38 participants, 12 dementia patients, 11 MCI patients, and 15 normal controls, recruited via the clinic in the Department of Neurology at Kyungpook National University Chilgok Hospital, Daegu, South Korea. We excluded patients with following conditions. (1) Patients with major psychiatric illnesses according to the criteria of DSM-IV, (2) patients who has been treated with chronic kidney disease, chronic respiratory disease, malignant tumor, and uncontrolled diabetes or hypertension. (3) Patients with systemic conditions that are known to cause dementia (e.g., hypothyroidism, vitamin B or folic acid deficiency, niacin deficiency, hypercalcemia, neurosyphilis, HIV infection), (4) Patients with substance-induced conditions, (5) patients with profound hearing loss, blindness, or gait disorder which makes it difficult to be tested^23^.

### Ethical considerations

This study was approved by the Institutional Review Board of Kyungpook National University (approval No. 2018-0149). All procedures about this study were explained to the participants and a written informed consent was obtained prior to participation. Patients with dementia and mild cognitive impairment provided their own written informed consent. Also informed consent was obtained from legally authorized representatives of the dementia and with mild cognitive impairment participants. The study was conducted following the principles established in the Helsinki Declaration update of 2008.

### Data collection

Demographic data, including age, sex, and educational level of the participants, were gathered, as displayed in Table 2. The disease duration of the patients was determined based on the date of diagnosis. The clinical diagnosis of patients was conducted by a clinician through the neuropsychological assessment^24^, which included K-MMSE, CDR, GDS, B-ADL, and I-ADL).

**Table 2:** Demographics and clinical characteristics.

Parameters	NC	MCI	Dementia	Overall F test	
				P value	Post-hoc
Age	69.1 ± 6.6	71.3 ± 9.0	76.2 ± 7.2	*P* < .001	a < c
Sex (M/F)	3/12	7/4	3/9	–	–
Education	8.4 ± 5.3	8.7 ± 4.3	9.8 ± 5.6	*P* = .78	–
Disease duration (year)	0	1.7 ± 1.8	3.0 ± 2.2	*P* < .001	a < b, a < c
K-MMSE	28.5 ± 1.5	25.5 ± 1.9	17.8 ± 3.8	*P* < .001	a < b, a < c, b < c
CDR	0.2 ± 0.3	0.5 ± 0.0	1.2 ± 0.4	*P* < .001	a < b, a < c, b < c
GDS	1.5 ± 0.5	3.3 ± 0.5	4.7 ± 0.5	*P* < .001	a < b, a < c, b < c
B-ADL	19.9 ± 0.3	19.4 ± 1.1	18.9 ± 1.9	*P* = .12	–
I-ADL	0.0 ± 0.0	0.4 ± 0.6	1.4 ± 0.7	*P* < .001	a < b, a < c, b < c

Mean ± standard deviations.

*NC* normal controls,* MCI* mild cognitive impairment, dementia: clinically demented peoples,* K-MMSE* Korean Mini-Mental State Examination, *CDR* clinical dementia rating, GDS global deterioration scale, *B-ADL* Barthel ADL index , *I-ADL* instrumental ADL index. Lowercase letters in the ‘post-hoc’ column refer to groups (a = NC, b = MCI, c = Dementia).

### Experimental, sensor-based ADL measurement system

#### Measuring devices

**Figure 3:**
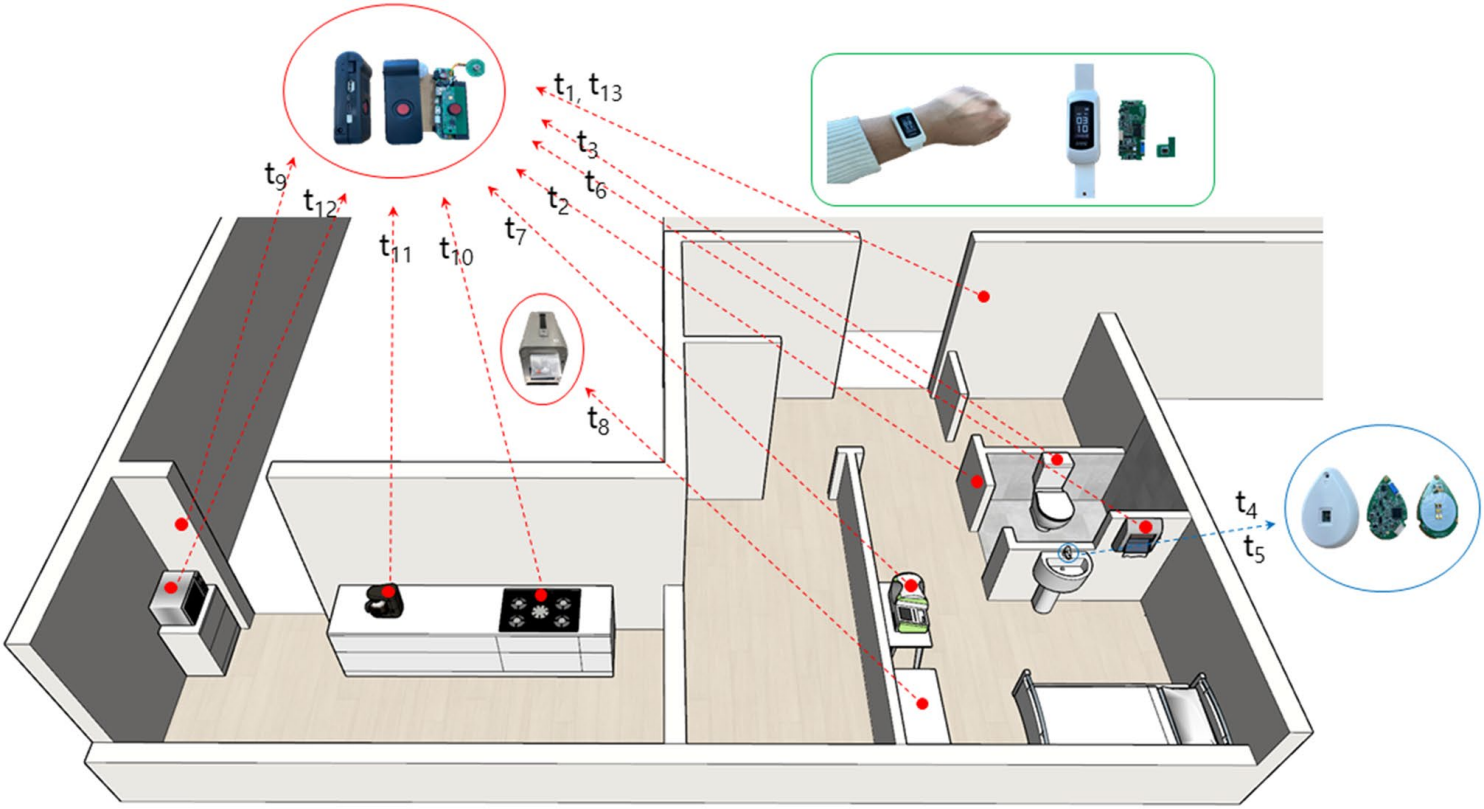
Illustration of the experimental devices and the ADL test environments. Red line; Stationary Resource Device, Blue line; External Sensor Device, Green line; Mobile identification Device (smart band). t1, t2…t13; tasks.

Three different devices were designed to measure and assess ADL in this experiment (Fig. 3). The resource device (RD), which was comprised of the stationary resource device (SRD) and the external sensor device (ESD), had a role in recognizing the activities of the participants and collecting data. The SRD was connected to a microwave, gas stove, Smart Pill Reminder, and a sphygmomanometer; then the device distinguished whether the participants indeed used each device or not, while the ESD controlled the sensors such as a gyro/accelerometer, passive infrared (PIR) sensors, and a frame sensor, for the actual measurements of the participants’ activities.

The mobile identification device (MID), wore by each participant, was developed in the form of Smart Band or Smart Tag. The MID communicated with the RD so that it could recognize a participant performing a specific task. The RD and MID used two different communication methods, low frequency (LF) and Bluetooth low energy (BLE). When a participant performed an experimental task, the LF signal was generated from the RD to wake up the nearby MID, and the BLE enabled the RD to gather the information regarding the participant’s identity and the distance from the MID. Furthermore, this combination of LF and BLE allowed the RD in every entrance to detect a participant’s access to the MID. In this study, we used this method to capture data on the movement of the participants tagged with the MID.

The location anchor hub (LAH) was placed in every unit space (10 × 10 cm) to collect data from the RD and MID in the same area and perform self-analysis of the data, whereby, it distinguishes the results of the tasks and decide whether it would repeat the recorded prompt or allow the participant to proceed to the next task. The LAH also gathered the received signal strength indicator of BLE, advertising from the MID so that it could broadcast a task-specific prompting, according to the presumed location of the MID. The LAH experiments automatically measure the ADL and analyze the results, including the task performance and task completion time based on the recorded data.

#### ADL measurement

Thirteen experimental tasks were developed for the measurement of the ADL (see Fig. 3). The performance of each task was recorded by a sensor-based platform at a general neurology ward in a university hospital. A recorded verbal prompting was broadcasted from the LAH before each task to assist the initiation of a task and to give a specific instruction regarding performing each task. All participants performed the same sequence of the tasks according to the verbal prompting. When they completed a task, regardless of the result was a success or failure, the prompting for the next task was broadcasted automatically.

A number was assigned to each task according to the sequence of the task performance. The procedure started from entering the test room (Task 1), followed by entering the toilet in the test room (Task 2). Then the participants were instructed to use the toilet (Task 3) and navigate to the sink (Task 4). After washing hands at the sink (Task 5) and wiping hands by a paper towel (Task 6), participants measured their own blood pressures with a sphygmomanometer (Task 7) in the test room. Subsequently, they were asked to use the Smart Pill Reminder, which provided pills automatically (Task 8). Next, participants were directed to the kitchen outside the test room (Task 9) and operate the gas stove (Task 10), followed by using the coffee machine (Task 11) and the microwave (Task 12) in the kitchen. Finally, they were asked to go back to the test room, where they initiated the experiment (Task 13). All the tasks except Task 5 were measured by the SRD. The ESD detected participants’ hands washing at the sink (Task 5). When a participant equipped with a MID initiated a sequence of the tasks, the measuring devices autonomously communicated with each other, and restored data, based on the participants’ location, the results of task performance, and task completion time in a memory device.

### Statistical analysis

Data preprocessing, data visualization, correlation analysis, and Linear Discriminant Analysis (LDA) was conducted by R software (version 3.6.3., 2020-02-29). We verified the relationship between the clinical ADL parameters (B-ADL, I-ADL) and thirteen ADL tasks using Spearman’s correlation coefficient (*rho*). Statistical Package for Social Sciences (SPSS Version 25, IBM) was used for LDA, a repeated-measured analysis of variance (ANOVA), and descriptive statistics. One-way ANOVA was used for participants’ demographics. The success proportion and task completion time were analyzed using repeated-measures ANOVA. Wilks’ lambda, Eigenvalues, and Discriminant hit ratio of three LDA models were calculated with SPSS. *P*-values for a two-tailed test were selected and *P* < 0.05, *P* < 0.01, and *P* < 0.001 were considered significant when comparing demographics, LDA, correlation, and ANOVA.
