# Author Correction: Clinical application of the experimental ADL test for patients with cognitive impairment: pilot study

**DOI:** 10.1038/s41598-021-88216-5

**Published:** 2021-04-13

**Authors:** Yong-Hyun Lim, Yookyeong Baek, Soon Ju Kang, Kyunghun Kang, Ho-Won Lee

**Affiliations:** 1 Center of Self-Organizing Software-Platform, Kyungpook National University, Daegu, South Korea; 2 Department of Neurology, School of Medicine, Kyungpook National University, 80 Daehakro, Bukgu, Daegu, 41566 Korea; 3 School of Electronics Engineering, College of IT Engineering, Kyungpook National University, Daegu, South Korea; 4 Brain Science and Engineering Institute, Kyungpook National University, Daegu, South Korea

Correction to: *Scientific Reports* 10.1038/s41598-020-78289-z, published online 11 January 2021

This Article contains errors in Reference 16 which is incorrectly given as:

Al-Jumaily, A. *et al.* Ambient assisted living technologies for older adults with cognitive and physical impairments: a review. *Eur. Rev. Med. Pharmaco.* **23**, 10470–10481 (2019).

The correct Reference 16 appears below as Reference [1].
